# The Research Progress of Magnesium Alloy Building Formwork

**DOI:** 10.3390/ma17143570

**Published:** 2024-07-18

**Authors:** Jinxing Wang, Zhicheng Wan, Jiaxu Wang, Yi Zou, Junyao Xu, Jingfeng Wang, Fusheng Pan

**Affiliations:** 1 College of Materials Science and Engineering, Chongqing University, Chongqing 400030, China; 202209021059@stu.cqu.edu.cn (Z.W.); 202309131157@stu.cqu.edu.cn (J.W.); 202209021040@stu.cqu.edu.cn (Y.Z.); junyaoxu@cqu.edu.cn (J.X.); jfwang@cqu.edu.cn (J.W.); fspan@cqu.edu.cn (F.P.); 2 National Engineering Research Center for Magnesium Alloys, Chongqing University, Chongqing 400030, China

**Keywords:** magnesium alloy, building formwork, research status, surface protection, chemical conversion

## Abstract

Building formwork is a kind of temporary supporting structure consumable material used in the construction field. In recent years, building formwork has gradually developed to become lighter, more environmentally friendly, and have higher performance. This sets higher requirements for the materials used to make building formwork. There is an urgent need to find a lighter and more durable material for building formwork. Magnesium alloys possess the advantages of low density, high alkali resistance, and high strength. As a building formwork material, it can reduce the weight of formwork and improve its durability. Therefore, a magnesium alloy is considered a material with high potential for building formwork. Currently, magnesium alloy building formwork has attracted the attention of many companies and research and development institutions, with preliminary research applications and good feedback on usage effects. It is highly possible to obtain the opportunity to put it into market application. However, to be applied on a large scale, there are still some important problems that need to be solved. These problems fall into three main areas, including the relatively low processing efficiency of magnesium alloy materials, the unstable price of magnesium alloys, and the fact that the formwork is easily corroded during storage. Firstly, at present, the main processing methods for magnesium alloy building formwork are casting and extrusion, and the production efficiency of both methods needs to be improved. Secondly, high-performance magnesium alloy materials are usually more expensive, which is not conducive to the large-scale application of the formwork. The price of magnesium alloys has fluctuated greatly in recent years, which increases the difficulty of promoting magnesium alloy building formwork. Thirdly, in the atmosphere, the oxide film on the surface of the magnesium alloy cannot play an effective role in corrosion resistance. So, surface treatment is necessary for magnesium alloy building formwork. Among the various surface treatment methods for magnesium alloys, the chemical conversion method has the advantages of being easy to operate, cost-effective, and having good corrosion resistance. It may be a very suitable protective method for large-scale applications of magnesium alloy building formwork and possesses excellent potential for application. The future of magnesium alloy building formwork will focus on new low-cost materials, high-efficiency processing technology, and low-cost green anti-corrosion technology. With in-depth research and the maturation of technology, magnesium alloy formwork is expected to play a more important role in the construction industry.

## 1. Introduction

Building formwork is a molding tool that allows concrete to solidify from a fluidic state to a solid state according to specified geometric dimensions [1]. Building formwork is consumed in large quantities annually as an essential building material. It is a critical consumptive material to ensure the quality, efficiency, safety, and economy of construction engineering. The global building formwork market was approximately USD 8.5 billion in 2023 and is expected to reach USD 11 billion by 2030. In recent years, the development of building materials worldwide has gradually tended towards lightweight materials, functionalization, and greening. People also put forward the requirements of lightweight development for building formwork. The lightweight development of building formwork can make the basic design of the building more economical and reasonable. Moreover, it can make building formwork more convenient in the transportation and installation process and play a role in improving efficiency. Lightweight construction formwork can also promote environmental protection, resource conservation, and technological innovation, aligning with a sustainable society’s requirements. With the advancement of technology and the enhancement in environmental awareness, the application prospects of lightweight building formwork will be broader.

The materials used for construction formwork directly affect the use effect and quality of the formwork. The cost of building formwork is also a significant factor affecting its application. Therefore, finding a lightweight and low-cost material that meets the performance requirements has become an important goal for the weight reduction in building formwork.

## 2. Application Status of Building Formwork

**Figure 1:**
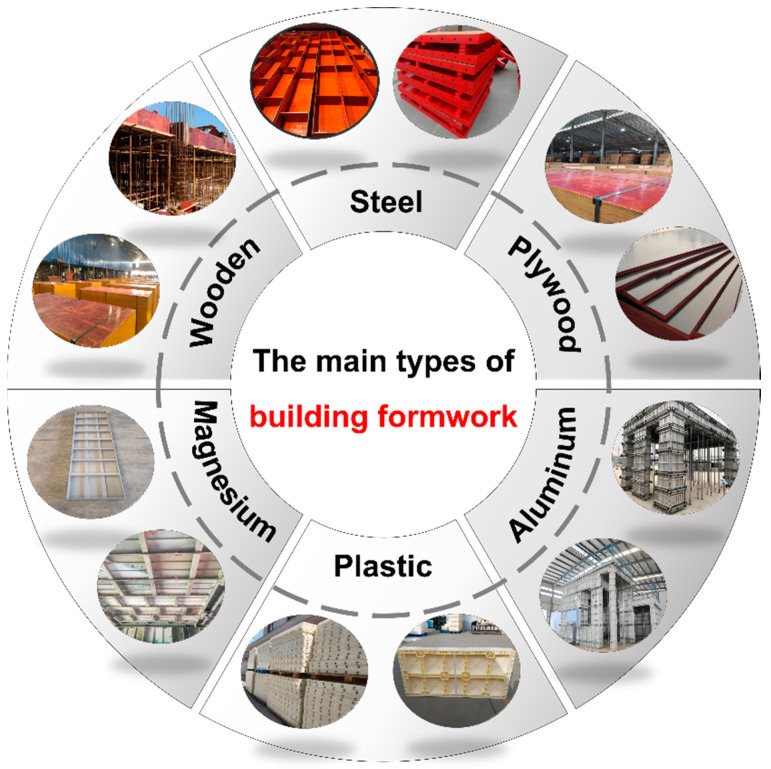
The main types of building formwork.

To meet the needs of a construction project, the materials of building formwork are constantly updated, and its performance is also continuously improved. At present, according to different materials, traditional building formwork is mainly divided into wooden formwork, plywood formwork, steel formwork, and aluminum alloy formwork. In recent years, some new types of formworks with great potential have emerged, for example, plastic formwork, magnesium alloy formwork, etc. The main types of building formwork are shown in Figure 1. 

Wooden building formwork occupies an essential position in the construction industry because of its advantages of low comprehensive cost and fast construction speed. Wood and plywood formwork account for up to 60% of the market. The raw materials of the wood formwork are easy to obtain and lightweight, which means it is easy to transport and install. This advantage reduces the handling cost and improves the construction efficiency. However, the shortcomings of the wooden formwork are also more apparent. It is easily damaged during use, and the turnover rate of wooden building formwork is low. This is undoubtedly a waste of wooden resources.

Plywood is a sheet material made up of a few layers, created by cutting logs into veneers or planing wood blocks into thin woods and then gluing them together with adhesives. Therefore, plywood can fully use the characteristics of different woods to produce a formwork with better performance. However, plywood also wastes wood resources, and it is easily affected by moisture and high temperatures during use. If not protected, it is easy for cracking and deformation to occur.

To protect the environment and improve economic efficiency, steel formwork entered the field of vision of the construction industry in the 1950s. At present, the market share of steel building formwork has reached 15%. The number of turnovers of the steel formwork is significantly higher than that of the previous wooden formwork, and the flatness is better. However, steel formwork can not only easily rust under acidic, humid conditions but also easily stick to concrete. In the construction process, it is also necessary to be equipped with corresponding lifting equipment and manual assistance. Based on the shortcomings of the above steel formwork, the use of steel building formwork has gradually reduced.

In recent years, aluminum alloys have become the main metal building formwork material, and their market share is 20%. As of 2023, the global aluminum alloy formwork market has reached 2,076,000 tons and is still growing. The number of aluminum alloy formworks is also increasing yearly and has reached 65 million square meters. Many scholars have conducted a series of studies on aluminum alloy building formwork, and there are many patents about aluminum alloy building formwork on the market [2,3,4,5]. Aluminum alloy formwork is known for its high strength and light weight. The aluminum oxide structure on the surface of aluminum alloys is particularly dense, offering good corrosion resistance in the atmosphere. The connections for aluminum alloy formwork typically use pins and wedges. It not only achieves seamless joining, enhancing the quality of the concrete surface, but also increases the speed of disassembly and assembly, reducing the construction period. Compared to plywood, there are higher turnover times and reuse rates in aluminum alloy formwork. They are also lighter than steel templates, allowing for manual handling and a simple, convenient assembly process without the need for mechanical assistance. However, the concrete used in construction is usually alkaline. The oxidation film on the surface of the aluminum alloy can easily react with the concrete, reducing the quality of the concrete surface and causing defects such as pitting. The poor corrosion resistance of aluminum alloy building formwork under alkaline conditions severely limits its application. This drawback has led scholars worldwide to seek out more suitable materials for construction templates.

Plastic building formwork is gradually being applied in the construction industry for its unique properties and advantages. This type of formwork is mainly made from plastic materials such as polypropylene (PP), polyethylene (PE), and polyvinyl chloride (PVC). These materials are usually characterized by abrasion resistance, corrosion resistance, and environmental protection. However, the strength and elastic modulus of plastic building templates are relatively small and cannot withstand large loads. The coefficient of thermal expansion and contraction of plastic formwork is relatively large, making it susceptible to temperature changes and potential deformation under extreme climatic conditions. Despite the many advantages of plastic construction formwork, the overall market recognition can still be improved and requires further standardization and regulation.

Lightweight development has become the main development direction of building formwork. With the development of technology and society, to improve the utilization rate of space, the demand for high-rise buildings is increasing. In the construction process of high-rise buildings, the transportation and installation of building formwork have become important factors affecting construction efficiency and safety. Therefore, lightweight building formwork has more advantages in the application of high-rise buildings. So far, aluminum building formwork has become the main metal building formwork. To further respond to the call for lightweight development, the building formwork industry will continue to develop in the direction of more efficiency, environmental friendliness, and economics. The application of new building formwork is gradually increasing. Some new building formworks, such as magnesium alloy and plastic, will bring new hope to the construction industry, provide more choices and possibilities, and show great application potential and market competitiveness.

## 3. Development Status of Magnesium Alloy Building Formwork

Magnesium alloys are the lightest metal structural material, with a density of only one-quarter of iron and two-thirds of aluminum. There are considerable advantages to using it as a lightweight material for building formwork. Moreover, magnesium alloys have good corrosion resistance under alkaline concrete conditions, which is conducive to improving the durability of formwork as a building formwork material. Magnesium alloys have been researched extensively as structural materials, and their mechanical properties have been greatly improved [6,7]. Although the magnesium alloy building formwork has not officially entered the market, magnesium alloys have gradually entered people’s development vision as a new building formwork material and shown considerable application prospects because of their qualified mechanical properties and good alkaline resistance.

### 3.1. Working Environment of Magnesium Alloy Building Formwork

Building formwork contacts with concrete directly during construction, and the acid–alkalinity of concrete has a certain influence on the performance and service life of formwork. The types of concrete commonly used in construction sites are mainly classified according to the strength level of concrete. Usually, the number after the cement grade represents the compressive strength of the concrete, such as C15, C20, C25, C30, C35, C40, etc. 

Concrete often appears alkaline because silicate minerals (such as tricalcium aluminate and dicalcium silicate) and aluminate minerals (such as tricalcium aluminate and quadalcium aluminate) begin to react chemically with water during cement hydration. The Ca(OH)_2_ they produce produces hydroxide ions (OH^−^) when dissolved in water, resulting in a pH of freshly mixed and hardened concrete typically between 12 and 13. 

The acid–base of concrete has an important effect on the formwork material. For example, the alkaline environment can provide a certain degree of protection for the steel bar against corrosion. For aluminum alloy building formwork, the alkaline environment will accelerate the corrosion of aluminum alloy surface film. Aluminum alloys can even react with concrete, affecting the molding quality of the concrete surface. For magnesium alloys, the good alkali resistance of magnesium alloys makes it easy to separate the magnesium alloy building formwork from the concrete, which is conducive to improving the forming quality of concrete. Therefore, common concrete is alkaline, which makes the magnesium alloy building formwork have a better advantage of alkali resistance.

### 3.2. Advantages of Magnesium Alloy Building Formwork

In recent years, the method of replacing aluminum alloys with magnesium alloys as building formwork materials to achieve the goal of being lightweight has received extensive attention, mainly due to the many advantages of magnesium alloys. First, the specific strength of magnesium alloys is high. The particular stiffness of magnesium alloys is on par with that of aluminum alloys and is much higher than that of plastics. This makes them suitable for structural parts in building formwork that require higher strength. This is thanks to the low density of magnesium, which is only 1.74 g/cm^3^, making it one of the lightest metals. Second, magnesium alloys have good corrosion resistance compared to aluminum under alkaline conditions. Aluminum is an amphoteric metal with poor corrosion resistance to acids and alkalis. Aluminum will react after contact with alkaline concrete, affecting the surface quality of the concrete. Magnesium alloys have good alkaline resistance. When the pH value is greater than 10.5, the Mg(OH)_2_ that forms on the surface of the magnesium alloy is very stable and can effectively slow down the corrosion of the magnesium alloy [8]. Thirdly, magnesium resources are abundant, and it is one of the most abundant elements in the world. Magnesium alloys may become a significant choice in construction engineering as iron and aluminum resources decrease. Fourthly, the recycling efficiency of magnesium alloys is high. After the end of their service life, magnesium alloy building formworks can be recycled. The casting process of magnesium alloys is relatively complicated. Because of the active chemical properties of magnesium, magnesium alloys often need to pay attention to protective gas and fire protection during casting. The recycling of magnesium alloys not only saves costs but also reduces resource depletion, which is beneficial for environmental protection. With the country’s continuous support for green building and new materials and the establishment of industry standards, magnesium alloy formwork is expected to become one of the mainstream products in the building formwork market.

### 3.3. Application Prospect of Magnesium Alloy Building Formwork

As an emerging building product, magnesium alloy building formwork is gradually gaining attention in the construction industry. Especially in the occasion of reducing the weight of the structure and improving the construction efficiency, the application of magnesium alloy building formwork can well meet the high requirements of construction companies and real estate developers for the performance, cost-effectiveness, and environmental protection of building products. The magnesium alloy building formwork industry is currently in the initial application and promotion stage. The market has reached a general consensus on the application prospects of magnesium alloy formwork.

In the world, the research of magnesium alloy building formwork is gradually deepening. In China, the market for magnesium alloy building templates is also gradually starting. In Shaanxi Province, the country’s first “Magnesium alloy building template research and development test production line” has been established. Some advanced magnesium alloy preparation technologies, such as pressure casting technology and rapid extrusion technology, have been introduced into the template preparation process. The corresponding cast magnesium alloy formwork and deformed magnesium alloy formwork have been manufactured. At present, many enterprises in China are involved in the magnesium alloy building formwork industry, such as Zhongtianshidai Magnesium Co., Ltd., Chongqing, China, Baowu Magnesium Technology Co., Ltd., Nanjing, China, etc. They actively opened up the application market of magnesium alloy building formwork. In addition, some scientific research institutions, such as the National Engineering Research Center for Magnesium Alloy Materials of Chongqing University, are also actively participating in the research and development of magnesium alloy formwork. At present, magnesium alloy building formwork products have been successfully tried in construction projects. The customer response and the market acceptance of magnesium alloy building formwork are good. The installation and operation method of these formworks is similar to that of aluminum alloy templates, and they are easy to use.

There are also challenges and opportunities in the application of magnesium alloy building formwork. The main challenges of the market include the relatively high production cost of magnesium alloy building formwork and the time required for the market to recognize and accept new materials. In terms of opportunities, with the advancement of technology and the expansion of production scale, the cost is expected to be reduced. The country’s policy support for green building materials and the growth of market demand will provide a broad development space for magnesium alloy building formwork. With the increasingly strict requirements for energy conservation and environmental protection in the construction industry, a series of policies have been introduced to encourage the development of green buildings and green building materials. These policies provide favorable conditions for the market promotion of magnesium alloy building formwork. Technological progress, increased market acceptance, policy support, and the improvement of industry standards are all driving the development of its market. In the future, with the establishment of industry standards and the improvement in market recognition, magnesium alloy building formwork is expected to occupy a more important position in the construction industry.

## 4. Problems in Magnesium Alloy Building Formwork

There are some problems in the research and development of magnesium alloy building formwork, such as the unstable price of magnesium alloys, low processing efficiency, and corrosion during storage. These problems seriously hinder the application of magnesium alloy building formwork. The next step is to accelerate the application of magnesium alloy building formwork from the aspects of cost control, processing technology, and surface treatment.

### 4.1. Unstable Cost Prices

**Figure 2:**
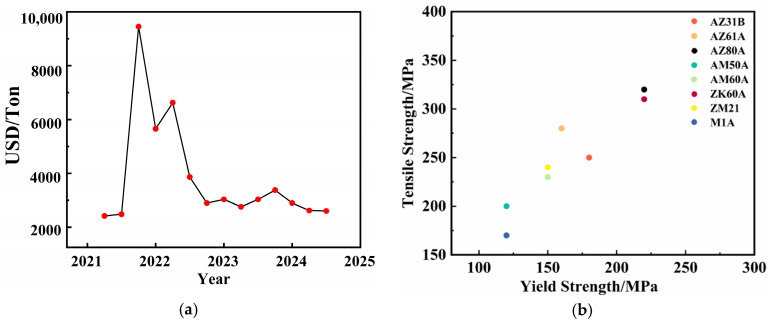
(**a**) Magnesium raw material prices from 2021 to 2024; (**b**) mechanical properties of magnesium alloy materials commonly used in building formwork.

In the past five years, the price of magnesium raw materials has experienced fluctuations and changes. The price of magnesium from 2021 to 2024 is shown in Figure 2a. Since 2021, the market price of magnesium has continued to grow, reaching a high of 10,192 USD/ton in September. In 2022, there was a significant decline in magnesium prices. At the beginning of the year, the average annual price of domestic raw magnesium was 5820 USD/ton, up 21.77% year-on-year. But then, the price continued to fall, and the lowest price in December was 3057 USD/ton. In 2023, the price of magnesium was still fluctuating, and the price of magnesium at the end of the year was 3071 USD/ton. As of 24 May 2024, the data show that 99.90% of the magnesium ingot factory cash offer including tax is 2657–2685 USD/ton. Through these data, we can see that the price of magnesium raw materials has experienced a trend of rising and then falling in the past few years, and the price has fluctuated greatly.

As a crucial temporary support structure in construction engineering, the building formwork material must possess certain properties to ensure its stability, safety, and reliability during the construction process. However, as a consumable part, the cost of magnesium alloy building formwork must also be taken into account simultaneously. The balance between the performance and cost of magnesium alloy building formwork is a focal point of concern.

Building formwork requires a certain degree of strength and rigidity to withstand the weight of the concrete and the loads that may be generated during construction. The mechanical properties of magnesium alloy materials commonly used in building formwork are shown in Figure 2b. To achieve this goal, the tensile strength of the formwork is generally between 150 and 350 MPa, and the yield strength should be within the range of 100–200 MPa. The performance of common magnesium alloys is rough, as shown in Table 1. From the table, it can be seen that the properties of common magnesium alloys have essentially met the requirements for use in building formwork.

For magnesium alloy building formwork, the commonly used magnesium alloy grades include AM50A, AZ31B, AZ61A, AM60A, AZ80A, ZK60A, ZM21, and M1A, among others. These grades of magnesium alloys are selected for different building formwork applications due to their respective properties. To reduce costs, some magnesium alloys with relatively lower performance and a tensile strength of 150–300 MPa, such as AZ31B, ZM21, and M1A, are used for building formwork to control costs. AZ80A, AZ61A, and ZK60A generally have a strength ranging from 300 to 400 MPa. They are widely used in building formwork that requires higher load-bearing capacity and better ductility due to their higher strength. To enhance the potential for application of building formwork, the economic aspect of the formwork should be reasonably considered, balancing the material properties with cost.

### 4.2. The Improvable Processing Efficiency

The method of processing is a crucial factor influencing the quality and performance of magnesium alloy building formwork. Currently, two main processing methods are primarily used for the preparation of magnesium alloy building formwork, and the production efficiency of both methods needs to be improved.

Die casting is one of the common methods for producing magnesium alloy building formwork. It involves injecting a molten magnesium alloy into a pre-designed mold and allowing it to cool and solidify under high pressure. This method can quickly and efficiently produce templates with complex shapes and precise dimensions. Die-cast magnesium alloy formwork is characterized by a smooth surface and uniform structure, making it suitable for mass production. However, magnesium alloys may experience shrinkage during the solidification process of die casting, which can lead to defects such as porosity and blowholes inside the castings. Especially when casting thick and large magnesium alloy workpieces, rapid cooling can easily cause thermal cracking, while slow cooling is not conducive to improving production efficiency. In the future, adopting advanced technologies such as the integrated die casting of magnesium alloys to improve processing efficiency will be a key focus of research.

Extrusion is a processing method that uses pressure to push magnesium alloy material through a die with a specific cross-sectional shape to obtain profiles with the desired shape and size. Extrusion is suitable for producing long lengths of magnesium alloy profiles with consistent cross-sections, such as the skeletal parts of beams and columns in building formwork. Due to factors such as the poor deformability of magnesium alloys and difficulties in temperature control during extrusion, the current extrusion speed of magnesium alloys is one-half to one-third of that of aluminum alloys. Additionally, the extrusion cost of magnesium alloys is relatively high. To enhance the market potential of magnesium alloy building formwork, developing rapid extrusion technology and improving production efficiency are important.

Advanced processing technologies of magnesium alloys are of great significance for promoting the use of magnesium alloy building formwork. Developing green die casting and rapid extrusion technologies in the future will be an effective means to improve the production efficiency of magnesium alloy building formwork. Enhancing the fluidity of magnesium alloys is also a way to improve processing efficiency. In addition, as a kind of building consumable, the processing cost of magnesium alloy building formwork should also be effectively controlled to popularize it widely. Overall, to promote magnesium alloy building formwork, its processing methods still need to become more efficient and cost-effective.

### 4.3. Protection Problems during Storage and Use

Magnesium alloys exhibit good corrosion resistance under alkaline conditions. The surface film on magnesium alloys is primarily composed of Mg(OH)_2_. The corrosion of magnesium alloys is essentially a process of electron transfer caused by their interaction with water. Once metal atoms are oxidized, they become metal ions. To maintain the electrical neutrality of the solution, a reduction reaction occurs to neutralize the free electrons released at the metal anode. Consequently, Mg^2+^ and OH^−^ combine to form Mg(OH)_2_ which deposits on the surface and forms a protective film. In humid air, the protective film on the surface of magnesium alloys becomes a double-layer structure, with the outer layer being Mg(OH)_2_ and the inner layer being MgO [9]. When MgO comes into contact with water, it becomes hydroxylated, forming a similarly loose and porous Mg(OH)_2_. There is an abundance of OH^−^ ions in alkaline solutions, so Mg(OH)_2_ can exist stably [10]. This implies that in high pH environments, Mg(OH)_2_ offers some protection to the substrate. The reaction formula of magnesium alloy surface film composition is as follows:

$${Mg=Mg}^{2+}+2e^{-}$$

$${2H_{2}O+2e^{-}=H}^{2}+2{OH}^{-}$$

$$Mg+2H_{2}O={Mg(OH)}_{2}$$

Mg(OH)_2_ produced on the surface of magnesium alloys can exist stably under alkaline conditions, and magnesium alloys have good alkaline resistance. However, in dry environments, the Mg(OH)_2_ film formed on the surface of magnesium alloys is loose and porous. The film is unable to fully encapsulate the magnesium alloy substrate, leading to poor corrosion resistance. At the same time, there are usually acidic gases such as CO_2_ and SO_2_ in the atmospheric environment, which will further accelerate the corrosion of the magnesium matrix. Therefore, magnesium alloy building formwork is prone to corrosion if there is no protective means during storage.

Cl^−^ is quite common in concrete and can accelerate the corrosion of magnesium alloys [11,12,13,14]. The good corrosion resistance of magnesium alloys under alkaline conditions makes them well suited as materials for construction formwork. However, the actual working environment of concrete is usually complex and diverse, where different types and concentrations of ions can also affect the corrosion resistance of magnesium alloys. Among these, the corrosion effect of Cl^−^ on magnesium alloys is the most significant. The impact of Cl^−^ on magnesium alloys is mainly twofold. First, the volume of Cl^−^ is relatively small. It can easily pass through the Mg(OH)_2_ protective film and reach the surface of the magnesium alloy to cause the matrix to rot. Second, the surface film of magnesium alloys will gradually fall off and thin under the continuous erosion of Cl^−^. Thus, the inherent surface film of magnesium alloys does not provide effective corrosion resistance against Cl^−^ [15]. Furthermore, as the concentration of Cl^−^ increases, the rate of corrosion of magnesium alloys also increases, with the reaction as follows:

$$Mg+{Cl}^{-}\to Mg{Cl}_{2}+2e^{-}$$

Scholars have focused on how to improve the corrosion resistance of magnesium alloys to Cl^−^. Various strategies have been proposed to address the corrosion resistance of magnesium alloys [16]. For example, chemical conversion coatings can form a dense protective film on the surface of magnesium alloys. It can prevent the erosion of chloride ions to a certain extent [17]. Plasma electrolytic oxidation (micro-arc oxidation) uses the instantaneous high-temperature sintering in the arc region to grow the ceramic layer in situ on the surface of the magnesium alloy matrix. The ceramic layer has the advantages of good corrosion resistance, strong bonding force, and high hardness. It can effectively improve the surface properties of magnesium alloys [18,19]. Electrochemical methods use electrode reactions to form protective coatings in the working environment, such as anodizing, electroplating, etc. [20]. These methods can form a dense oxide or other compound film on the surface of magnesium alloys. It can effectively isolate the contact between chloride ions and the magnesium alloy matrix, thereby improving the corrosion resistance of chloride ions. Laser modification technology uses high-energy lasers to treat the surface of magnesium alloys to form an alloyed surface layer. This way can improve the wear resistance of magnesium alloys and upgrade their protection against chloride ions [21].

In summary, magnesium alloys have excellent alkali resistance, making them an ideal material for new types of construction formwork. In the working environment, to obtain a longer service life of building formwork, it is useful to use clamps and brackets made of the same magnesium alloy. However, to enhance the corrosion resistance of magnesium alloy building formwork during storage and use, surface treatment is needed. The surface treatment methods used for construction formwork should be simple, convenient, and cost-effective. Yet, most surface treatment methods for magnesium alloys require a strict environment and conditions for operation. This is undoubtedly unfavorable for the industrial application of magnesium alloy construction formwork. How to improve the corrosion resistance of magnesium alloys during storage and use has become an urgent problem in the application of magnesium alloy building formwork, and it is also one of the hot spots in the research of magnesium alloys.

## 5. Chemical Conversion Surface Protection Technology of Magnesium Alloy Building Formwork

In recent years, researchers have dedicated efforts to improving the corrosion resistance of magnesium alloys in a variety of environments [22,23]. Anti-corrosion technology for magnesium alloy building formwork must be economically viable and simple to implement. Common anti-corrosion techniques such as electroplating, micro-arc oxidation, vapor deposition, and spraying are effective but involve complex processes. Chemical conversion is a process in which a metal reacts with a chemical conversion solution and then forms a film layer with good adhesion on the metal surface. The chemical conversion coating turns the alloy surface into a passive state, isolating it from the corrosive medium. Because of its simple operation, stable performance, suitability for large-scale production, and low cost, chemical conversion technology is considered to be a potential anti-corrosive technology suitable for magnesium alloy building formwork.

### 5.1. Chromate Chemical Conversion Coating

Early research on chemical conversion coatings for magnesium alloys mainly concentrated on chromate conversion films [24]. Chromate conversion coatings possess excellent corrosion resistance and self-healing properties, making them widely used in various industries [25,26]. Dichromate chemical conversion films are generally considered to possess good corrosion resistance in humid air. In the process of forming chromate chemical film, soluble hexavalent chromium ions are reduced to insoluble trivalent chromium ions to cover the surface of magnesium alloys, thus preventing the further corrosion of magnesium alloys. Some of the residual chromium ions in the film also possess a self-healing function, which can effectively improve the quality of the film and slow down the corrosion of magnesium alloys.

However, the solution of chromate chemical coating contains hexavalent chromium ions which have strong carcinogenicity and biological toxicity. The transformed liquid is not only harmful to the human body and environment but also makes it difficult to dispose of waste liquid. This shortcoming limits the development and application of chromate coatings. At present, hexavalent chromium ions have been banned from commercial use. Research on the chemical conversion coating of magnesium alloys has gradually developed towards chromium-free coatings [27,28,29,30,31,32].

### 5.2. Phosphate Chemical Conversion Coating

To replace chromate conversion coating that may impact human health and the environment, people have turned to low-cost and low-toxicity phosphate conversion coating (PCCs) [33]. The quality, density, and corrosion resistance of PCCs are affected by pH, temperature, phase, microstructure, alloy composition, pretreatment method, bath composition, additive type, and soaking time [34,35]. The generation of PCCs is roughly a two-step process. In the first step, when the magnesium matrix is immersed in the phosphoric acid solution, magnesium is dissolved as the anode, and H^+^ is reduced to hydrogen at the cathode. This process is accompanied by a rise in pH. In the second step, due to the rise in the pH value, the solubility of metal phosphate decreases. The precipitation will then precipitate and gradually deposit on the magnesium matrix to form a phosphate conversion coating. The good adhesion of PCCs ensures a certain level of corrosion resistance. In recent years, the development of the construction formwork industry has made phosphate chemical conversion film coatings more eye-catching. However, some of the main reasons for the lack of the industrial application of magnesium alloy phosphate chemical conversion films are the existence of cracks, the uneven growth of protective films on the substrate, and the lack of self-healing properties [36].

The phosphate coating needs pretreatment before preparation. The effect of pretreatment can be roughly divided into changing surface roughness and surface activity. For example, there is a great relationship between the surface roughness and the quality of phosphate coatings. A too smooth coating will reduce the nucleation site and binding force, and one too rough will increase the porosity of the film [37,38]. Zhang et al. [39] studied the influence of different pretreatment processes on the quality of the phosphate conversion coating of the AZ91 magnesium alloy. It is more beneficial to form a dense conversion film after polishing than sandblasting. The surface roughness of the sample after sandblasting is higher and results in an uneven and microporous microstructure which further leads to more severe galvanic corrosion. Therefore, suitable roughness is significant for the quality of phosphate conversion coatings. Li et al. [40] compared the effects of oxalic acid, phosphoric acid, and colloidal Ti activators on the morphology and corrosion resistance of the phosphate conversion coating formed. They found that all three preactivators increased the specific surface area to varying degrees, thus shortening the nucleation time, increasing the nucleation amount, and reducing the nucleus size. Samples pretreated with phosphoric acid have the best corrosion resistance, which may be related to factors such as the preactivator changing the pH and roughness of the magnesium alloy surface.

**Figure 3:**
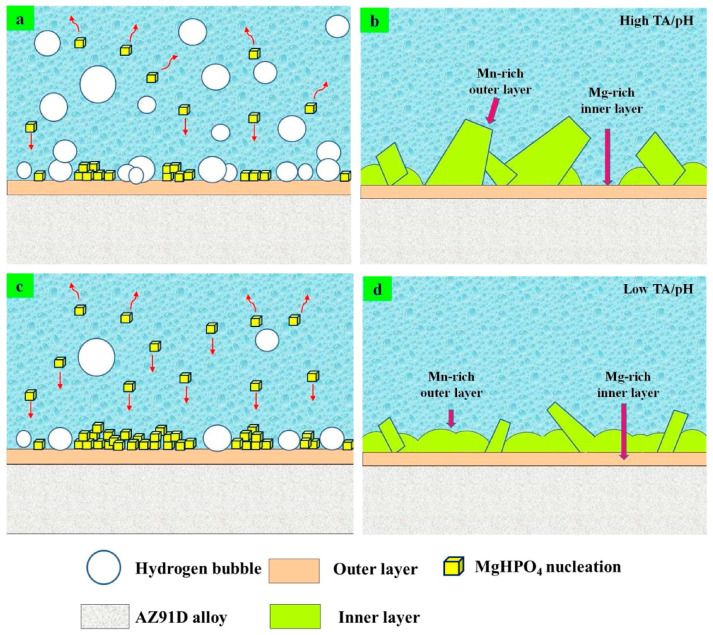
A schematic illustration of the formation mechanisms and microstructure of the PCC-coated (The red arrow shows the direction of MgHPO_4_). (**a**) Mg alloy AZ91D specimens in the baths with high TA/pH 26.9; (**b**) Mg alloy AZ91D specimens in the baths with high TA/pH 30.5; (**c**) Mg alloy AZ91D specimens in the baths with high TA/pH with low TA/pH 15.2; (**d**) Mg alloy AZ91D specimens in the baths with high TA/pH 19.1 [52].

Due to the prominent corrosion resistance problem of magnesium alloy building formwork, scholars have studied the corrosion resistance of phosphate conversion coatings in concrete environments [41]. Han et al. [42] prepared a black chromium-free phosphate chemical conversion coating on AZ91D. Compared with other coatings, magnesium alloy samples coated with phosphate chemical conversion coatings possessed excellent corrosion resistance in 3.5 wt% NaCl solution. Zhou et al. [43] also prepared a phosphate coating with good corrosion resistance on AZ91. After analysis, it was found that the film was a compound phosphate of Mg and Al and showed obvious passivation characteristics. Van et al. [44] compared the corrosion properties of zinc phosphate and magnesium phosphate chemical conversion coatings on AZ31. It was found that magnesium phosphate possessed better corrosion resistance in a salt spray environment. Fu et al. [45] prepared a composite conversion coating with Mg(OH)_2_, magnesium phosphate, and manganese phosphate using a one-step immersion treatment. Compared with the bare magnesium alloy, the corrosion resistance of the coating in the environment containing Cl^−^ was significantly improved. Lee et al. [46] studied the corrosion resistance of permanganate in a phosphate salt bath to the prepared coating. It was found that after adding permanganate, the coating was thinner and denser. The cracks in the coating were fewer, and the samples showed stronger corrosion resistance. Yuan et al. [47] prepared a zinc phosphate chemical conversion coating on the surface of AZ61, followed by hydrothermal treatment. After hydrothermal treatment, ZnO and stearic absorbent layers were grown on the coating, with almost no cracks and superhydrophobicity. Zhou et al. [48] studied the phosphate conversion coating on the surface of the AZ91D magnesium alloy in a manganese phosphate dihydrogen bath and evaluated the effect of Ca^+^ addition on the microstructure of the coating. The coating containing Ca^+^ showed crystal characteristics, and the addition of Ca^+^ increased the corrosion potential and enhanced the corrosion performance of the coating on Cl^−^. However, the coating containing Ca^+^ is not uniform, and the grain size is different, which harms the forming of the conversion film. Liu et al. [49] also used the chemical conversion method to prepare a CaHPO_3_·2H_2_O coating on the AZ91D surface. It was found that the coating has a foliaceous microstructure. NH_4_H_2_PO_4_ acts as both an acidifier and an activator, and the entire coating preparation process takes only 20 min at room temperature. Wang et al. [50] prepared a CaHPO_3_ coating with good corrosion resistance on the surface of the AZ41 magnesium alloy with the aid of ultrasound. Ultrasonic assistance makes phosphate conversion film forming more uniform and helps to eliminate the limitation of workpiece size by the hydrothermal method [51]. The protection of CaHPO_3_ film makes the sample have good corrosion resistance in the environment containing Cl^−^. However, there were still a few cracks on the surface of the generated conversion film. Preparing a crack-free protective film is the key to further research. Zhang et al. [52] proposed the total acidity/pH value (TA/pH) for the first time with the help of thermodynamic equilibrium calculation to guide the chemical formulation design of phosphate-based conversion coatings on magnesium alloys. The formation mechanism and microstructure of the PCC-coated magnesium alloy AZ91D sample in the bath are shown in Figure 3. In the solution with high TA/pH, the growth rate of the membrane is high, and it is easy to form coarse particles and partially uncovered areas. In contrast, the nucleation density in a solution with low TA/pH is small, and it is easy to promote PCC and refined particles. Therefore, a low-TA/pH solution is more conducive to the preparation of phosphate conversion coatings with uniform microstructure and improving the corrosion performance of magnesium alloys.

### 5.3. Vanadate Chemical Conversion Coating

**Figure 4:**
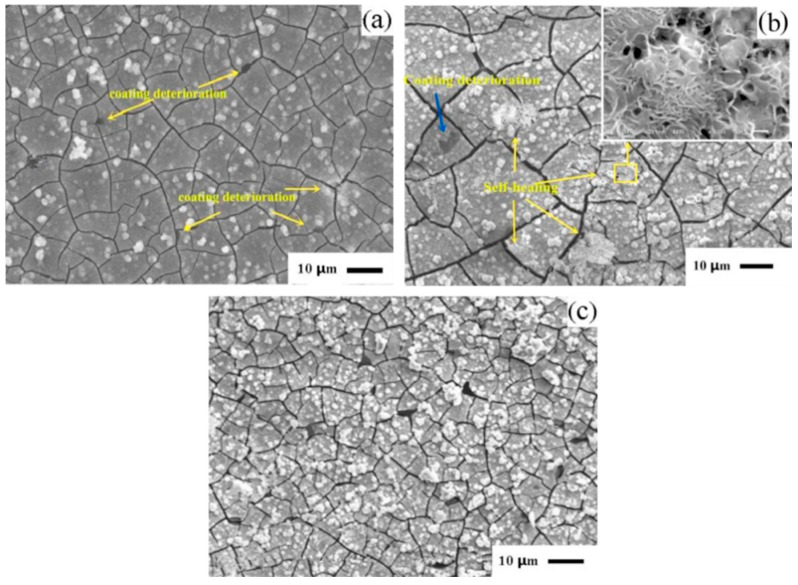
The Ce–V conversion coating after (**a**) 12 h, (**b**) 72 h. (The yellow square is the amplification area) and (**c**) 168 h of immersion in 3.5 wt% NaCl solution [58].

As an alternative to chromate conversion coating, vanadate conversion coating has attracted the attention of scholars [53,54]. Hamdy et al. [55] found that the continuity of the vanadate coating prepared in 50 g/L vanadium solution was better than that in 10 g/L and 30 g/L. However, the increase in pH will reduce the corrosion resistance of the film. The formation of vanadium oxide in flower form can effectively resist the corrosion of Cl^−^ on the magnesium matrix. They also observed some self-healing ability in the vanadate coating [56]. Niu et al. [57] found that the vanadate coating formed in the vanadium phosphate solution of 4–5.5 g/L of NaVO_3_ possessed a three-dimensional network structure. Among them, the vanadate coating formed in the vanadium phosphate solution of 4 g/L of NaVO_3_ was the densest. In addition, when the content of NaVO_3_ in the vanadate solution was 4 g/L, not only was the microstructure of the conversion paint the finest, but also the adhesion and corrosion resistance of the electrophoretic paint were the best. Jiang et al. [58] found that after adding cerium nitrate, pentavalent vanadium in the vanadate coating was reduced to tetravalent vanadium. An appropriate amount of cerium nitrate makes the coating thicker and the CeVO_4_ layer more compact. Figure 4 shows the evolution of coating morphology over soaking time. In 3.5 wt% NaCl solution, the coating shows good corrosion resistance with a certain self-healing ability.

Yang et al. [59] prepared a V/Ce conversion coating on AZ31B. The coating was relatively fractured and scattered with spherical particles. Compared with the matrix magnesium alloy, the corrosion potential of the sample increased from −1.506 V to −1.306 V. The barrier effect of coating and the increase in corrosion potential increased the corrosion resistance of the magnesium alloy. Feng et al. [60] studied the corrosion inhibition effect of the pH value of vanadate solution on the AZ31 magnesium alloy. The pH value of the converted solution was 5.0, which produced a thin film dominated by tetravalent vanadium. The films containing mainly trivalent vanadium were produced at pH values higher than 7.7. Feng et al. [61] evaluated the ability of various pairings of NaVO_3_, Na_3_PO_4_, Na_2_HPO_4_, and NaF to delay AZ31 corrosion. Among them, the inhibitory effect of Na_3_PO_4_-NaVO_3_ was the best. This provides an idea for making a process based on phosphate–vanadate conversion coating. Vanadate solution can also be used as a pretreatment of a cerium-containing conversion coating to improve the corrosion resistance of magnesium alloys. Lee et al. [62] used vanadate solution instead of toxic HF to prepare a chemical conversion coating containing cerium. After treatment in vanadate solution, the growth rate of the cerium-containing conversion coating was significantly slowed down, and the quality of the coating was improved. Cerium can also be used to improve the corrosion resistance of vanadate coatings. Lo et al. [63] studied the effect of sodium metavanadate on the corrosion resistance of a cerium coating. After adding NaVO_3_ to the conversion solution, the size density of bubbles in the coating was significantly reduced, and the corrosion resistance was improved.

### 5.4. Stannate Chemical Conversion Coating

As an alternative to chrome-containing ion coating, stannate chemical conversion coating is also a non-toxic metal salt coating [64]. The formation of a stannate chemical conversion coating is largely dependent on the dissolution of magnesium. Basic stannates nucleate around large amounts of Mg^2+^, mainly around the β phase [65]. Stannate chemical conversion coating has the characteristics of low cost and low toxicity, which are suitable for magnesium alloy building formwork.

**Figure 5:**
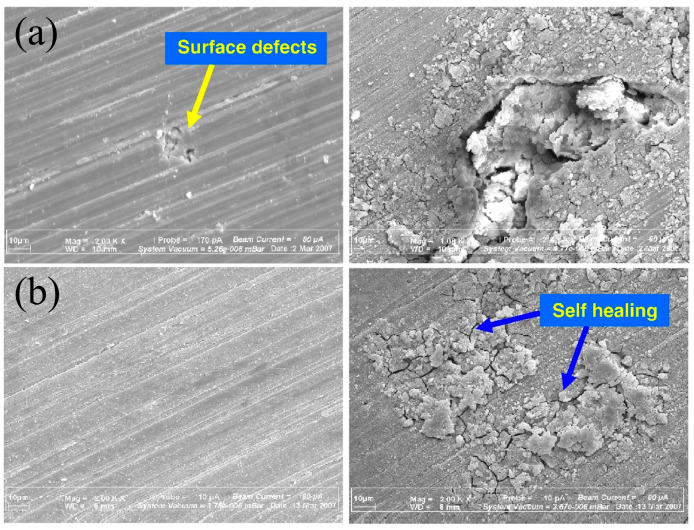
SEM images of the samples after 7 days in NaCl solution. (**a**) Untreated; (**b**) treated [70].

Elsentriecy et al. [66] prepared a stannate chemical conversion coating on the AZ91D surface. They found that stannate accelerated the dissolution of magnesium and promoted the deposition of the conversion film during the conversion process. The corrosion resistance of the magnesium alloy was improved by the stannate conversion coating. In a further study, they pickled the surface of AZ91D with hydrochloric acid, hydrofluoric acid, and their mixed solution to explore the effect of this measure on the corrosion resistance of the prepared stannate conversion layer [67]. After pickling the mixed solution of hydrochloric acid and hydrofluoric acid, the surface of the magnesium alloy was dissolved relatively uniformly. The density and corrosion resistance of the protective film were improved. Zucchi et al. [68] found that compared with the permanganate/phosphate bath, there were no interconnected pores in the stannate conversion layer produced by the stannate bath, and the corrosion resistance was also better. Lin et al. [69] studied the effects of solution composition and temperature on the microstructure and corrosion resistance of the stannate conversion coating on the AZ61 magnesium alloy. Increasing the concentration of stannate ions and decreasing the pH value of the solution can not only reduce the size of coating particles but also increase particle density. It is conducive to the formation of the best corrosion resistance of the conversion coating. At the same time, due to the shortest soaking time in the best chemical conversion reagent, this also facilitates the convenience of coating preparation. Hamdy et al. [70] studied the corrosion resistance of stannate conversion coatings in an environment containing Cl^−^. Samples that were simply soaked in stannate solution possessed the best corrosion resistance, with a polarization resistance four to five times higher than uncoated samples. Figure 5 shows the surface of different samples after soaking in 3.5 wt% NaCl solution for 7 days. The samples showed some self-repair after treatment, while serious cracks, pitting, and micro-cracks were observed on the surface of other untreated samples.

Li et al. [71] studied the composite conversion coating of rare earth cerium and stannate for the first time. It was found that a single coating can improve the corrosion resistance of magnesium alloys. The composite coating composed of cerium oxide, magnesium stannate, and magnesium hydroxide had the best corrosion resistance.

Stannate chemical conversion coating has the advantages of low cost and low toxicity, which are conducive to its application in the field of magnesium alloy building formwork. However, its limited corrosion resistance often makes it necessary to use it in combination with other coating or surface treatment technologies. Further research on a better formulation and pretreatment process of stannate chemical conversion solution is the key to improving its corrosion resistance.

### 5.5. Molybdate Chemical Conversion Coating

Molybdate chemical conversion coating has attracted the interest of many scholars because of its excellent corrosion resistance and environmental friendliness. It is well known that Mo(VI) has the effect of slowing down corrosion because of its strong oxidation. It can stabilize the reduction product and form a passivation film. Like chromate, molybdate and its adsorbed products can effectively slow down corrosion and resist Cl^−^ [72,73]. Molybdate chemical conversion coating has excellent corrosion resistance, but it is expensive. In the future, under the premise of reducing the cost, ensuring the corrosion resistance of molybdate conversion coating is the focus of its research.

The molybdate conversion coating prepared by the chemical conversion method of Ishizaki et al. [74] was mainly composed of Mg(OH)_2_, MoO_2_, MoO_3_, and MoF_2_. Compared with AZ31, the electrode potential of the coating was lower, and the corrosion current was smaller. Mu et al. [75] prepared a chromium-free composite coating using a solution containing molybdate and cerium nitrate. The coating was mainly composed of MoO_3_, MoO_2_, CeO_2_, Ce_2_O_3_, MgO, and Mg(OH)_2_. Compared with the AZ91 matrix, the coated sample has a better corrosion resistance, better corrosion potential, and lower corrosion current. Wang et al. [76] prepared a molybdate coating on Mg-8.5Li alloy. By adjusting the concentration of ammonium molybdate solution and adding potassium permanganate, it was found that the resulting coating was still better than bare alloys and some chromate conversion coatings, despite some cracks. The corrosion resistance of the coating prepared by adding potassium permanganate decreased. Accelerating the deposition of molybdenum on the surface of the magnesium alloy can reduce the cracks on the coating surface. Yang et al. [77] studied the effect of NaF and La(NO_3_)_3_ on the corrosion resistance of molybdate conversion coating. After the action of NaF and La(NO_3_)_3_, the deposition rate of molybdate ions accelerated, and the coating particles were spherical. The resulting coating was more uniform, and the corrosion resistance was better. Farahat et al. [78] carried out the alkaline etching of AZ31D with KOH before preparing the molybdate conversion coating. They found that the corrosion resistance of the sample was significantly enhanced. After alkaline etching, the microstructure of the coatings produced on AZ31D showed flower-like and needle-like networks. These structures can effectively prevent the corrosive medium from reaching the metal matrix, thereby improving the resistance to pitting and crevice corrosion. This simple pretreatment method can effectively improve the corrosion resistance of magnesium alloys to Cl^−^. Zhu et al. [79] carried out the micro-arc oxidation of a molybdate coating in a NaSiO_3_-dominated solution. Micro-arc oxidation is beneficial to the formation of MgAl_2_O_4_, and a new phase MoSi_2_ was formed. The corrosion potential of the enhanced molybdate coating produced by micro-arc oxidation was more right, and its corrosion resistance was better. Xu et al. [80] prepared a Mo-Sn-W ternary metal conversion coating by the one-step chemical conversion method. The microstructure of Mo-Sn-W@TMSCC is hemispherical, and its corrosion resistance is higher than that of Mo, Sn, and W binary metal conversion coatings. Mo-Sn-W@TMSCC can effectively prevent the corrosion of Cl^−^, and it takes 192 h for complete failure in 3.5 wt% NaCl solution. Yong et al. [81] prepared a composite coating of molybdate and phosphate. It is composed of metaphosphate and molybdenum oxide, and its corrosion resistance is comparable to that of traditional chromate coatings. However, due to the complex operation process, it is difficult to realize the industrial application of the coating.

### 5.6. Permanganate Chemical Conversion Coating

Scholars have studied permanganate conversion coatings with less environmental impact to replace toxic chromium-containing coatings [23,25,82]. The outstanding corrosion resistance of permanganate conversion coating makes it possess certain potential in the field of magnesium alloy building formwork. However, the permanganate chemical conversion solution is unstable, and the manganese ion slightly harms the environment. 

Umehara et al. [83] produced coatings containing manganese oxide as early as 2001. Although the coating produced at that time was still amorphous, the corrosion resistance was comparable to that of some chromate coatings. Subsequently, they obtained a protective film from a permanganate salt bath consisting mainly of manganese oxide, magnesium oxide, and magnesium hydroxide [84]. The film’s corrosion resistance was the same as the conversion film containing chromium but thinner. To pursue better corrosion resistance, people began to use permanganate conversion coatings together with other conversion coatings. Zhao et al. [85] prepared a composite coating with good adhesion using a phosphate/permanganate solution. Although there were some small holes on the surface, the corrosion resistance of the sample was better than that of some chromium-containing coatings. Lin et al. [86] studied the microstructure evolution of a permanganate/phosphate composite coating on the AZ31 alloy. It was found that the coating temperature had a significant effect on the coating composition, and the growth rate of the coating was reduced by increasing the solution temperature. In addition to the temperature parameter, Mosialek et al. [87] also evaluated the influence of the concentration of the conversion solution and conversion time on the corrosion resistance of the prepared coating. They obtained the best parameters for the preparation of the permanganate/phosphate composite coating in the experiment.

**Figure 6:**
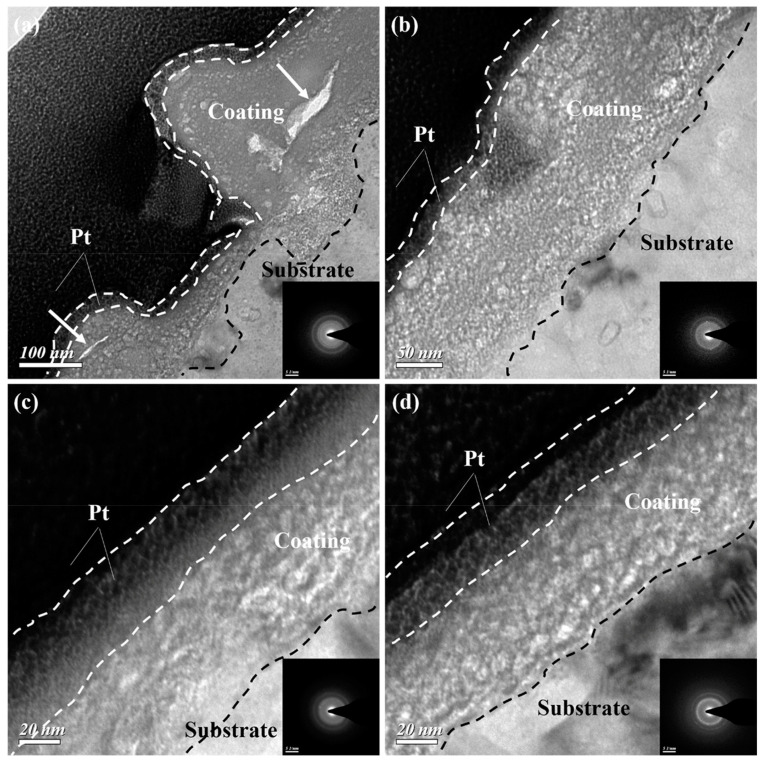
Cross-sectional TEM characterization of the permanganate conversion coating formed after 10 s of immersion in the permanganate conversion bath at (**a**) 15, (**b**) 25, (**c**) 40, and (**d**) 60 °C [89].

Yang et al. [88] studied the effects of fluoride ions on the microstructure and corrosion properties of a permanganate coating on the AZ91D alloy. The study found that fluoride ions can delay the formation process of the coating, so the coating is more uniform, and the thickness is smaller. There were no obvious cracks on the surface of the permanganate coating formed in the fluorine-containing solution. Hung et al. [89] studied the growth kinetics of a permanganate coating on the LZ91 alloy at 15–60 °C. As shown in Figure 6, there was a more uniform coating formed at 60 °C. The increase in temperature reduced the growth rate and thickness of the film and hardly affected the composition of the conversion film. Therefore, the corrosion resistance of permanganate conversion film at 40 °C and 60 °C was better than that at 15 °C and 25 °C. Jian et al. [90] found that the corrosion resistance of the permanganate coating formed in potassium permanganate solution with cerium ions was better than traditional permanganate conversion film. The presence of cerium ions promotes the formation of a dense coating, resulting in fewer cracks.

## 6. The Development Direction of Magnesium Alloy Building Formwork in the Future

As a new type of building material with multiple advantages, magnesium alloy building formwork will develop in a multifaceted direction in the future. This includes the development of alloy materials, improvement in processing technology, research on surface treatment technology, establishment of industry standards, and so on. With the support of relevant policies and the advancement of the industry, it is expected that magnesium alloy building formwork will gain wider application in the construction field.

To enhance the performance of magnesium alloy building formwork, the improvement in alloy materials is one of the future research directions. Magnesium alloy formwork needs to further increase its strength and durability while maintaining its lightweight nature to meet the construction industry’s demand for the higher strength and durability of formwork. At the same time, attention should be paid to the demand for new building materials in the international market, and products adapted to different regions should be developed.

Improving die-casting and extrusion processes can reduce the production cost of magnesium alloy formwork, making magnesium alloy building formwork more competitive in the market. It can also improve production efficiency and shorten the time to market, allowing for a rapid response to market changes. Continuous improvement in integrated die-casting and high-speed extrusion processes is also one of the research directions. Efficient processing methods can further enhance the application potential of magnesium alloy building formwork.

In the future, the surface treatment technology for magnesium alloy building formwork will strive to reduce costs and improve the economic competitiveness of magnesium alloy formwork. Appropriate surface treatment is needed to enhance the corrosion resistance of magnesium alloy formwork. Among the many surface treatment technologies for magnesium alloys, chemical conversion is considered suitable for magnesium alloy building formwork. There is a need in the future to find more economical and effective chemical conversion solution systems and to further improve the chemical conversion process.

Cooperation with construction companies and research institutions will be necessary to expand the scale of engineering practice with magnesium alloy building formwork and to promote its application in a broader range of fields. The development of magnesium alloy building formwork requires not only support from materials and technology but also collaborative efforts along the entire industry chain. Close cooperation among all stages, from raw material supply to product manufacturing, construction application, and recycling, will help promote the efficient operation and sustainable development of the entire industry chain.

In summary, the direction of development for magnesium alloy building formwork is diverse. Its development will depend on the efforts and progress in technological innovation, cost control, market development, and other aspects. With the development of the global economy and the advancement of the construction industry, the magnesium alloy formwork industry is expected to usher in an even broader development prospect.

## 7. Conclusions

As a temporary support structure, building formwork is an essential construction material. Building formwork has been increasingly trending towards being lightweight and environmentally friendly in recent years. Magnesium alloy building formwork, with its characteristics of being lightweight, high-strength, and alkali-resistant, has shown great potential for application in the construction industry. Currently, the research and development of magnesium alloy building formwork also face some challenges, such as improving the corrosion resistance of magnesium alloys, reducing production costs, increasing production efficiency, and meeting the performance requirements of construction work. To address these challenges, researchers and companies are continuously exploring new solutions. In the future, the market competitiveness of magnesium alloy building formwork should be improved by adopting more efficient surface treatment technologies, developing high-performance magnesium alloy materials, and optimizing production processes. Among the various surface treatment technologies for magnesium alloy building formwork, chemical treatment has shown unique advantages due to its economic and convenient characteristics. Developing economical and durable chemical conversion films is key to enhancing the corrosion resistance of magnesium alloy building formwork and promoting its application. Overall, the future development of magnesium alloy building formwork should focus on improving corrosion resistance, optimizing processing technology, and reducing overall costs. With the concept of environmental protection and sustainable development becoming more deeply rooted in the minds of people, magnesium alloy building formwork is expected to see a continuous increase in market demand. As a green building material, magnesium alloy building formwork will be used more and more widely in the construction industry.

## Figures and Tables

**Table 1:** Properties of common magnesium alloys.

Density	Tensile Strength	Yield Strength	Elongation	Stiffness	Corrosion Resistance
1.75–1.85 g/cm^3^	150–400 MPa	100–300 MPa	5–20%	50–100 HB	alkali-resisting

## Data Availability

Not applicable.
